# Synthesis and Preliminary Evaluation of a Novel ^18^F-Labeled 2-Nitroimidazole Derivative for Hypoxia Imaging

**DOI:** 10.3389/fonc.2020.572097

**Published:** 2021-02-02

**Authors:** Jing Lu, Chi Zhang, Xi Yang, Xi-Juan Yao, Qun Zhang, Xin-Chen Sun

**Affiliations:** ^1^ Department of Radiation Oncology, The First Affiliated Hospital of Nanjing Medical University, Nanjing, China; ^2^ Department of Health Promotion Center, The First Affiliated Hospital of Nanjing Medical University, Nanjing, China; ^3^ Department of Radiation Oncology, Fudan University Shanghai Cancer Center, Fudan University, Shanghai, China; ^4^ Department of Oncology, Shanghai Medical College, Fudan University, Shanghai, China

**Keywords:** PET tracer, hypoxia, 2-nitroimidazole, chemoradiotherapy resistance, ^18^F

## Abstract

**Objective:**

Hypoxia is prevalent in tumors and plays a pivotal role in resistance to chemoradiotherapy. ^18^F-MISO (^18^F-labeled fluoromisonidazole) is currently the preferred choice of PET hypoxia tracers in clinical practice, but has severe disadvantages involving complex labeling methods and low efficient imaging due to lipophilicity. We aimed to design a novel nitroimidazole derivative labeled by ^18^F *via* a chelation technique to detect hypoxic regions and provide a basis for planning radiotherapy.

**Materials and Methods:**

First, we synthesized a 2-nitroimidazole precursor, 2-[4-(carboxymethyl)-7-[2-(2-(2-nitro-^1^H-imidazol-1-yl)acetamido)ethyl]-1,4,7-triazanonan-1-yl]acetic acid (NOTA-NI). For ^18^F-labeling, a ^18^F solution was reacted with a mixture of AlCl_3_ and NOTA-NI at pH 3.5 and 100°C for 20 min, and the radiochemical purity and stability were evaluated. Biological behaviors of Al^18^F-NOTA-NI were analyzed by an uptake study in ECA109 normoxic and hypoxic cells, and a biodistribution study and microPET imaging in ECA109 xenografted mice.

**Results:**

Al^18^F-NOTA-NI required a straightforward and efficient labeling procedure compared with ^18^F-MISO. The uptake values were distinctly higher in hypoxic tumor cells. Animal studies revealed that the imaging agent was principally excreted *via* the kidneys. Due to hydrophilicity, the radioactivities in blood and muscle were decreased, and we could clearly distinguish xenografted tumors from para-carcinoma tissue by PET imaging.

**Conclusions:**

The nitroimidazole tracer Al^18^F-NOTA-NI steadily accumulated in hypoxic areas in tumors and was rapidly eliminated from normal tissue. It appears to be a promising candidate for hypoxia imaging with high sensitivity and resolution.

## Introduction

Hypoxia associated with tumor resistance to chemoradiotherapy has been reported to occur in more than 60% of solid tumors. The unlimited proliferation of tumor cells and the abnormal distribution of neoplasm vasculature give rise to oxygen supply-demand disequilibrium. Hypoxia appears in tumor cells that are beyond the range of effective oxygen diffusion (about 100-200 μm from nutrient vessels) (1). A hypoxic microenvironment induces a series of self-protection mechanisms, such as apoptosis inhibition, cell-cycle arrest, increased anaerobic glycolysis and tumor angiogenesis (2). Furthermore, hypoxia, an independent prognostic risk factor, triggers resistance to radiotherapy, such that the lethal radiation dose in hypoxic cells is 3-fold higher than that in normoxic cells, which contributes to recurrence and metastasis by reactivating quiescent cells (3–5). Re-oxygenation should play a crucial role in individualized anti-tumor treatment. Hypoxia imaging by positron emission tomography (PET) combined with computed tomography (CT) or magnetic resonance (MR) is a non-invasive method that could be used to measure the level of hypoxia in tumor areas and outline the biological target volume (BTV) to boost the radiation dose. Furthermore, the variance in the standardized uptake value (SUV) of hypoxic regions before and after treatment could be used to evaluate the treatment efficacy and predict the prognosis.

Although many PET tracers that are specifically designed to detect hypoxia have been developed, including nitroimidazole labeled by ^18^F, ^68^Ga or ASTM combined with a Cu (^62^Cu or ^64^Cu) (6–10), ^18^F-MISO (^18^F-labeled fluoromisonidazole) is currently the most common hypoxia imaging agent used in a clinical setting. Nitroimidazole may undergo single-electron reduction by xanthine oxidase. In normoxic cells, the nitro group can be reoxidized and washed out. However, under hypoxic conditions, it is further reduced to form highly reactive intermediates that can bind to cellular macromolecules and be irreversibly trapped in hypoxic cells (11). Due to its lipophilicity, ^18^F-MISO rapidly penetrates both tumors and normal tissues to provide a robust and reproducible signal, but this also causes slow clearance kinetics and a low target-to-background ratio and contrast (12, 13). Furthermore, ^18^F-labeling requires time-consuming reactions such as repeated evaporations, radiolabeling, purification and complex synthesis conditions that include a high temperature and the presence of a base catalyst.

We aimed to synthesize a novel hypoxia imaging agent to overcome these drawbacks. McBride et al. reported a high-efficiency labeling method in which ^18^F is first attached to aluminum as Al^18^F, which is then bound to a chelate attached to a foundational structure (14). This one-pot process requires only 15 min without a heating evaporation step, and has been used for labeling with positron-emitting nuclides like ^18^F and ^68^Ga (15, 16). We used this proven method to design a PET hypoxia tracer with a stable structure and high hydrophilicity, where 1,4,7-triazacyclononane-1,4,7-triacetic acid (NOTA) as a chelate joins Al^18^F and nitroimidazole.

## Materials and Methods

### Synthesis of NOTA-NI

1) *tert-butyl 2-(2-nitro-^1^H-imidazol-1-yl)acetate (II)*

A mixture of 2-nitro-^1^H-imidazole (I, 1 g, 8.8 mmol), tert-butyl 2-bromoacetate (1.8 g, 9.2 mmol) and potassium carbonate (1.3 g, 8.8 mmol) in dry acetonitrile (20 ml) was refluxed under nitrogen for 3h. The reaction mixture was filtered, and the filtrate was evaporated. The residue was purified by recrystallization using ethyl acetate/hexane to give the product as a white solid. Yield: 1.4 g (71%).

2) *N-(2-bromoethyl)-2-(2-nitro-^1^H-imidazol-1-yl)acetamide (IV)*

A solution of compound II (1.32 g, 5.8 mmol) in CH_2_Cl_2_ (39 ml) and Tallow Fatty Acid (TFA, 17 ml) was stirred at room temperature for 3.5 h. After the reaction was completed (as determined by thin layer chromatography (TLC)), the filtrate was evaporated, and the residue was used in the next step without purification. A mixture of 2-(2-nitro-^1^H-imidazol-1-yl)acetic acid (III), 2-bromoethanamine hydrobromide (1.1 g, 5 mmol), 2-(7-azabenzotriazol-1-yl)-N,N,N’,N’-tetramethyluronium hexafluorophosphate (HATU, 2 g, 5.2 mmol) and N,N-diisopropylethylamine (DIEA, 5 ml) in dry CH_2_Cl_2_ (200 ml) and dry N,N-dimethylformamide (DMF, 20 ml) was stirred at room temperature overnight. After the reaction was completed (as determined by TLC), the filtrate was evaporated, and the residue was dissolved in 50 ml of water. The aqueous layer was extracted with ethyl acetate (50 ml × 3). The organic layers were collected and washed with 5% aqueous K_2_CO_3_, 5% aqueous HCl and saturated brine. The organic layer was dried over MgSO_4_. The crude product was purified by silica gel column chromatography (petroleum/ethyl acetate = 1:2) to give the pure product as a solid. Yield: 0.52 g (32%).

3) *1,4-bis(tert-butoxycarbonylmethyl)-1,4,7-triazanonane (VI)*

A solution of tert-butyl bromoacetate (21.4 g, 55mmol) in CHCl_3_ (100 ml) was slowly added to triazacyclononane (V, 6.5 g, 50 mmol) in CHCl_3_ (50 ml) over 1 h using a syringe pump. The resulting mixture was stirred at room temperature for 24 h. The reaction mixture was then filtered, and the filtrate was evaporated. The residue was treated with water (30 ml), and the resulting solution was adjusted to pH 3 using 1 M HCl and washed with ether (20 ml × 3). The aqueous layer was then adjusted to pH 11 using 1 M NaOH and extracted with CH_2_Cl_2_ (20 ml × 3). The organic layers were collected and evaporated, and dried to obtain a crude product. Hexane (15 ml) was added to give the disubstituted product as a solid. Yield: 10.9 g (61%).

4) *tert-butyl 2-{4-[2-(tert-butoxy)-2-oxoethyl]-7-[2-(2-(2-nitro-^1^H-imidazol-1-yl)acetamido)ethyl]-1,4,7-triazanonan-1-yl}acetate (VII)*

A solution of compound IV (665 mg, 2.4 mmol) in dry acetonitrile (7 ml) was added dropwise to a mixture of compound VI (715 mg, 2mmol) and potassium carbonate (331 mg, 2.4 mmol) in dry acetonitrile (10 ml). The reaction was stirred at room temperature for 24 h. The mixture was filtered, and the filtrate was evaporated. The residue was purified by silica gel column chromatography (CH_2_Cl_2_/MeOH = 9:1) to give the purified product as an oil. Yield: 354 mg (32%).

**Figure 1:**
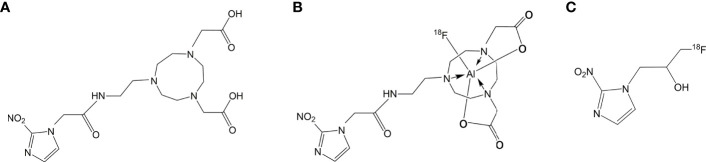
**(A)** Structures of NOTA-NI. **(B)** Structures of Al^18^F-NOTA-NI. **(C)** Structures of ^18^F-MISO.

5) *2-[4-(carboxymethyl)-7-[2-(2-(2-nitro-^1^H-imidazol-1-yl)acetamido)ethyl]-1,4,7- triazanonan-1-yl]acetic acid (NOTA-NI)* (**Figure 1A**).

A solution of compound VII (354 mg, 0.64 mmol) in trifluoroacetic acid (2 ml) was stirred at room temperature for 4.5 h. The reaction mixture was concentrated to dryness in vacuo and treated with ether (10 ml), and the ether layer was decanted. The residue was dissolved in water (2 ml) and washed with CHCl_3_ (2-5 ml). The aqueous layer was concentrated to dryness in vacuo to provide the pure product (254 mg, 90%). ^1^H NMR (D_2_O, 500 MHz): δ 7.37 (d, 1H, J = 1.5 Hz), 7.16 (d, 1H, J = 1.5 Hz), 5.36 (s, 1H), 5.18 (s, 2H), 3.75 (s, 4H), 3.65 (t, 2H, J = 6.0 Hz), 3.51 - 3.53 (m, 4H), 3.45 (t, 2H, J = 6.0 Hz), 3.35 (m, 4H), 3.18 (s, 4H). ^13^C NMR (MeOD, 500 MHz): δ 172.8, 167.3, 145.1, 128.0, 127.1, 54.9, 54.3, 51.6, 50.7, 49.9, 35.4.

### ^18^F-Labeling

H_2_^18^O was bombarded by protons in a cyclotron to produce a ^18^F aqueous solution that was collected under a positive pressure of helium. The radiation dose of fluoride ions was 20 mCi measured by a CRC-15R activity meter (CAPINTEC, Florham Park, NJ).

A stock solution of AlCl_3_ (0.005mg) prepared by dissolving AlCl_3_·6H_2_O in AcONa buffer (pH 3.5) and Glacial acetic acid (10 μL) was added to 0.1 mg of the synthesized NOTA-NI in acetonitrile (200 μL), and specific amounts of glacial acetic acid, aluminum trichloride, and acetonitrile were added. The prepared ^18^F solution (50 μL) was then added to the solution of a precursor. The reaction mixture was placed on a 100°C heating block for 20 min. Labeled compounds were passed through an Alumina-N light cartridge (prewashed with 10 ml of normal saline) to remove unlabeled Al^18^F, and washed with normal saline (5 ml). The collected labeled products were heated to evaporate the rest of the solution and redissolved in saline. The radioactive product was analyzed by analytical HPLC and no impurity was found (**Figure 1B**).

### Stability Study

The stability of the above Al^18^F-NOTA-NI in phosphate-buffered solution (PBS) at room temperature and in human serum at 37°C was analyzed by instant thin-layer chromatography-silica gel (iTLC-SG) [80% methyl cyanide (MeCN) in water] at approximately 0, 3 and 6 h.

### *In Vitro* Cell Uptake Study

Cell uptake studies were carried out using the ECA109 cell line (esophageal squamous cell carcinoma, ESCC). The cells were seeded into 24-well plates at a density of 1 × 10^5^ cells per well before overnight incubation in RPMI-1640 culture medium enriched with 10% fetal bovine serum, and then preincubated under normoxic or hypoxic (1.0 ± 0.1% pO_2)_ conditions for 4 h. In the test group, Al^18^F-NOTA−NI was added to the wells (about 0.15 MBq/well) and cells were incubated for 10, 30, 60, 90 and 120 min (n = 3 per time point). The hypoxia tracer of control group was ^18^F-MISO (**Figure 1C**) with the same dosage. Wells were then washed with RPMI-1640, and cells were isolated by tryptic digestion and dissolved in PBS. The cell uptake value was measured using a γ counter.

### Biodistribution Study in Xenografted Mice

ECA109 cells were grown in a normal environment. Cells were harvested after treatment with trypsin and washed with 10 ml of PBS by centrifugation (3000 rpm). Four-week-old female BALB/c mice were each injected subcutaneously with 2 × 10^5^/0.1 ml cancer cells in the right shoulder. At 14 days after the induction of tumor xenografts, all mice had developed a solid tumor mass (weight approximately 1.0 g) in which hypoxia essentially built up. Each xenografted mouse in the test group was administered Al^18^F-NOTA-NI (1.85 MBq/0.1 ml) *via* a tail vein, while each one in the control group was injected ^18^F-MISO with the same dosage. Mice were sacrificed by decapitation at 20, 60 and 120 min after radiotracer administration (n = 3 per time point). Tumor, muscle and significant organs were excised and weighed, and blood samples were taken. Counts were obtained using a γ counter. The radioactivity contents of representative organs are expressed as percentages of injected dose per gram of tissue (% ID/g). Results are shown as the mean and SD for four animals. The study was approved by the ethics committee of Nanjing Medical University (IACUC-1712027).

### MicroPET Study of Xenografted Mice

MicroPET scans were performed using an Inveon microPET scanner (Siemens Medical Solutions, Erlangen, Germany). Al^18^F-NOTA-NI (3.7 MBq/0.1 ml) was administered *via* tail vein injection to xenografted mice after isoflurane anesthesia. Pimonidazole (80 mg/kg), a kind of classical hypoxia probe, was simultaneously injected *via* a tail vein. Two-hour dynamic imaging was performed by acquiring 12 × 10-min frames from the start of tracer injection to measure time-activity curves (TACs) (n = 4). Ten minutes of static microPET images were acquired at 30, 60, 120 and 240 min after injection (n = 4 per time point). Image reconstruction was performed by Fourier rebinning using an ordered subsets expectation maximization (OSEM) algorithm without attenuation or scatter correction. Regions of interest (ROIs) over the tumor, normal tissue, and major organs were drawn on decay-corrected whole-body coronal images. The radioactivity concentration was obtained from mean pixel values within the multiple ROI volumes. Imaging ROI-derived % ID/g was calculated by dividing the ROIs by the administered activity. Subsequently, animals were sacrifice and tumors were excised, snap frozen, and embedded in ornithine carbamyl transferase. Sections of 8-μm thickness were prepared for fluorescein isothiocyanate (FITC) studies to verify the intratumoral hypoxia. Sections were exposed to FITC-conjugated murine antipimonidazole monoclonal antibody diluted 1:25 for one hour at room temperature, and then imaged again with the markers visualized by red fluorescence.

### Statistical Analysis

Quantitative data are expressed as mean ± SD. Means were compared using one-way ANOVA and Student’s t-test. For all hypothesis testing, we used two-sided *p*-values <0.05. The results were compared with the data obtained using ^18^F-MISO under the same experimental conditions.

## Results

### Physicochemical Evaluation

**Figure 2:**
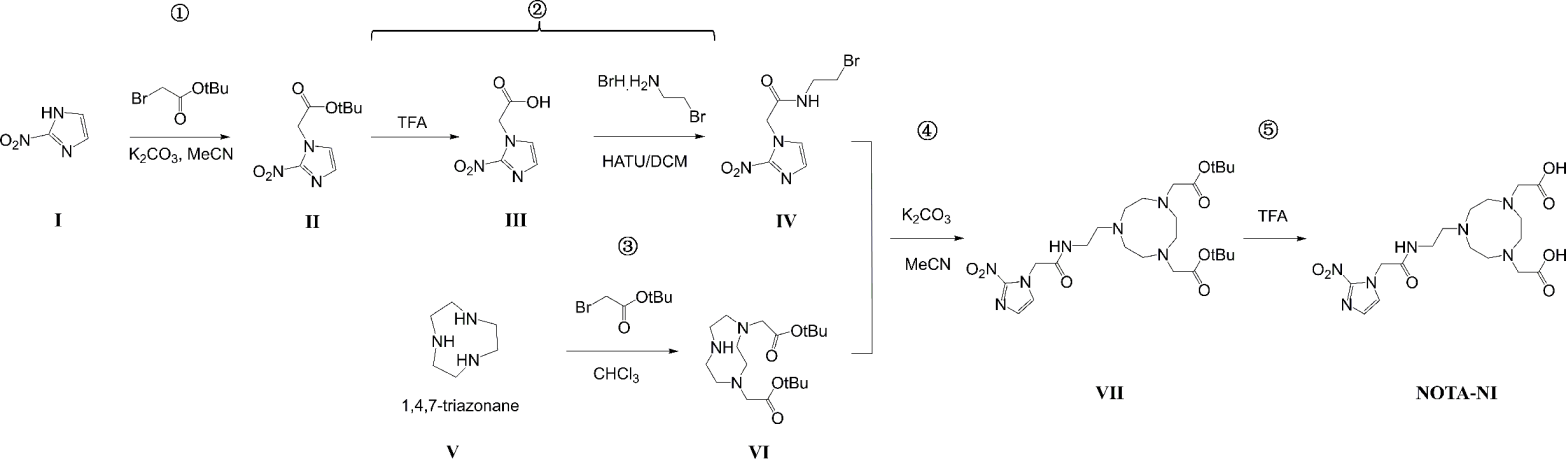
Synthesis pathways of NOTA-NI, a 2-nitroimidazole derivative.

**Figure 3:**
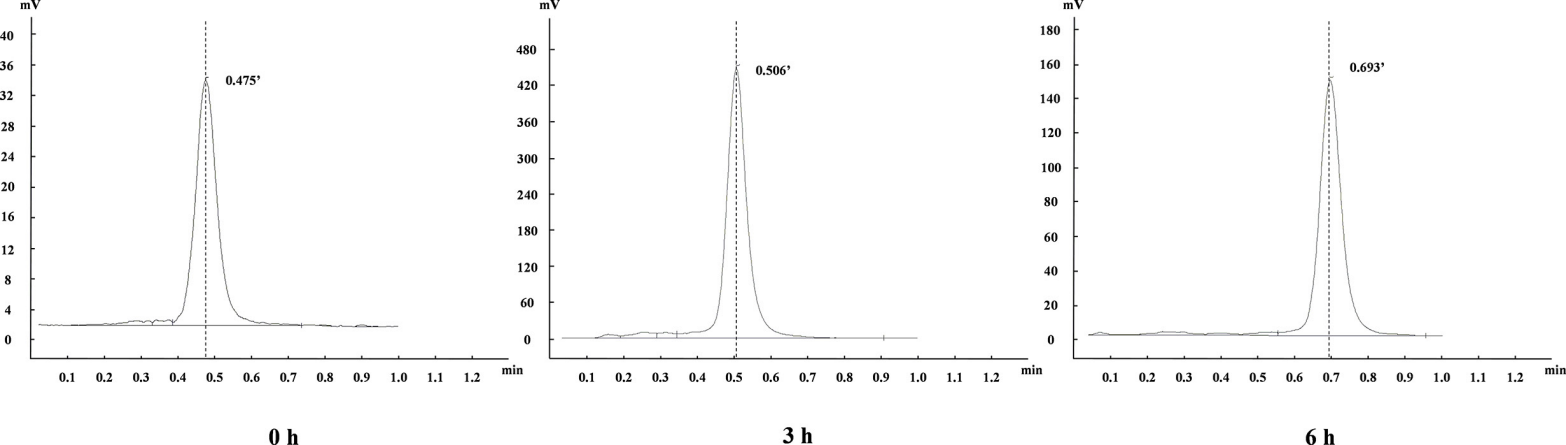
Radiochemical purities determined by iTLC-SG at 0, 3 and 6 h.

The chemical structure of NOTA-NI is described in the Methods and the synthetic process is summarized in **Figure 2**. The reaction time for precursor labeling was shortened to 20 min with only one step under 100°C heating (labeling rate, 52.6 ± 3.7%). Al^18^F-NOTA-NI (after purification by HPLC) is a colorless transparent liquid that is significantly more hydrophilic (logP: -0.952 ± 0.034, pH 7.4) than ^18^F-MISO (logP: -0.353 ± 0.016, pH 7.4) to reduce the duration of concentration in normoxic cells. The specific activity of either the novel compound or ^18^F-MISO was 50 GBq/μmol and radiochemical purity was more than 95%. In the *in vitro* stability study, more than 99% of the radiochemical purity of Al^18^F-NOTA-NI in PBS was retained for 6 h (**Figure 3**). Meanwhile, in human serum at 37°C, more than 95, 85, and 80% of the radiochemical purity was retained after incubation for 0, 3 and 6 h, respectively. This good stability ensures adequate transportation and detection *in vivo*. Considering ^18^F is a nuclide with the short half-life, we suggest that new imaging agents should be applied in four hours after labeled by ^18^F.

### Cell Uptake Study

**Figure 4:**
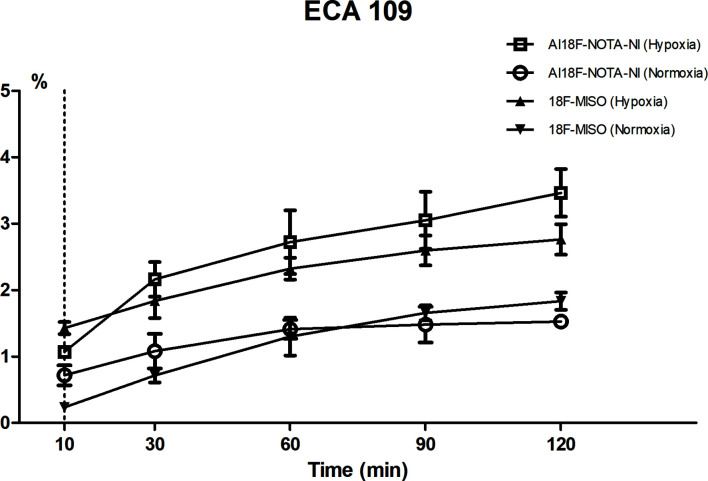
Cell uptake of Al^18^F-NOTA-NI under hypoxic and normoxic conditions (n = 4 per time point).

Cell uptake *in vitro* was studied using the cell line ECA109. As shown in **Figure 4**, the uptake rate of Al^18^F-NOTA-NI under hypoxic conditions gradually reached a peak value of 3.46 ± 0.56% at 120 minutes after administration, which was 2.27-fold higher than that under normoxia. The hypoxic-to-normoxic uptake ratio of Al^18^F-NOTA-NI was increased by 1.53-fold compared to that of ^18^F-MISO (P < 0.05) (**Table 1**).

**Table 1:** Hypoxia/normoxia uptake ratios of Al^18^F-NOTA-NI and ^18^F-MISO (*p* < 0.05).

Agent	Uptake Rate (mean ± SD, N = 3)
Al^18^F-NOTA-NI	2.268 ± 0.353
^18^F-MISO	1.533 ± 0.182

### Biodistribution Study

**Figure 5:**
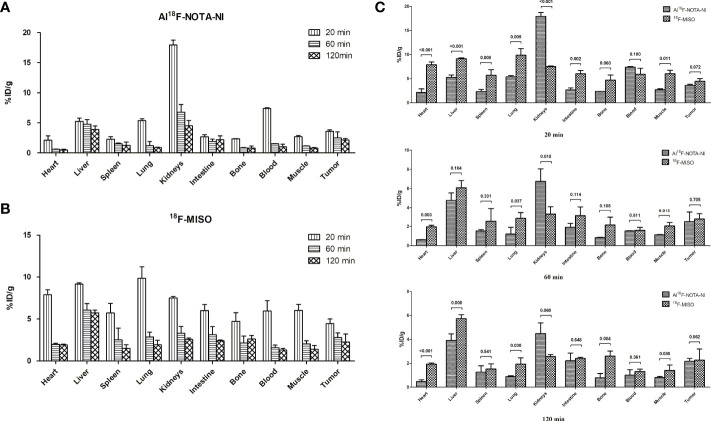
**(A)** Percentages of injected dose per gram of tissue (%ID/g ± SD) in major organs of xenografted mice at 20, 60, and 120 min p.i. for Al^18^F-NOTA-NI (n = 3 per time point). **(B)** Percentages of injected dose per gram of tissue (%ID/g ± SD) in major organs of xenografted mice at 20, 60, and 120 min p.i. for Al^18^F-NOTA-NI (n = 3 per time point). **(C)** Uptake value (%ID/g ± SD) of Al^18^F-NOTA-NI in comparison with that of ^18^F-MISO in the same kind of organ at 20, 60, and 120 min p.i (n = 3 per time point).

For both Al^18^F-NOTA-NI and ^18^F-MISO, a biodistribution study was performed at different time points (20, 60 and 120 min) after intravenous injection of these labeled derivatives into mice bearing ECA109 xenografts (**Figures 5A, B**). **Figure 5C** and **Table 2** show the uptake values in tumor, blood, muscle and other normal tissues as well as the tumor-to-blood (T/B) and tumor-to-muscle (T/M) uptake ratios for Al^18^F-NOTA-NI and ^18^F-MISO. For Al^18^F-NOTA-NI, the highest uptake was observed in the kidneys (17.90 ± 0.81%ID/g) at 20 min post-injection (p.i.), and this value rapidly decreased to 4.50 ± 0.89%ID/g at 120 min, indicating that this drug is principally excreted *via* the kidneys. The next highest initial uptake was in blood, and again the activity rapidly decreased over time (7.38 ± 0.14%ID/g at 20 min and 1.01 ± 0.44%ID/g at 120 min). Initial uptake in the liver was similar to that in blood (5.24 ± 0.54%ID/g at 20 min p.i.), but the activity remained fairly stable (4.76 ± 0.78%ID/g at 60 min and 3.90 ± 0.55%ID/g at 120 min). In tumor tissue, the initial uptake promptly reached (3.61 ± 0.22%ID/g at 20 min), and moderately declined over time (2.51 ± 0.99%ID/g at 60 min and 2.16 ± 0.24%ID/g at 120 min). ^18^F-MISO showed a higher initial uptake in the liver, intestine and muscle compared to Al^18^F-NOTA-NI. Normal organs showed slower depuration, and the specific activities in the liver and kidneys at 120 min p.i. were 5.74 ± 0.33%ID/g and 2.57 ± 0.16%ID/g, respectively. According to the literature, ^18^F-MISO is excreted through both the urinary and hepatobiliary tracts (15). Although tumor had a higher uptake of ^18^F-MISO, more drug penetrated into surrounding soft tissues and blood at the same time point. T/B and T/M of Al^18^F-NOTA-NI gradually increased, and were apparently higher at 120 min than those for ^18^F-MISO (T/B: 2.32 ± 0.53 vs. 1.70 ± 0.39, *p* = 0.253; T/M: 2.67 ± 0.08 vs. 1.58 ± 0.24, *p* = 0.004). Statistically significant differences between T/M of the two agents could probably be attributed to the rapid clearance of the highly hydrophilic Al^18^F-NOTA-NI.

**Table 2:** Organ uptake, tumor uptake, tumor/blood ratio and tumor/muscle ratio of Al18F-NOTA-NI and 18F-MISO.

Organ (Mean ± SD, %ID/g)	20 min			60 min			120 min		
	Al^18^F-NOTA-NI	^18^F-MISO	*p*-value	Al^18^F-NOTA-NI	^18^F-MISO	*p*-value	Al^18^F-NOTA-NI	^18^F-MISO	*p*-value
Liver	5.24 ± 0.54	9.16 ± 0.16	<0.001*	4.76 ± 0.78	6.08 ± 0.76	0.104	3.90 ± 0.55	5.74 ± 0.33	0.008*
Intestine	2.67 ± 0.37	2.67 ± 0.37	0.002*	1.91 ± 0.40	1.91 ± 0.40	0.114	2.21 ± 0.65	2.41 ± 0.10	0.648
Liver+ Intestine	7.91 ± 0.84	15.16 ± 0.57	<0.001*	6.67 ± 1.02	9.21 ± 0.74	0.025*	6.11 ± 0.98	8.14 ± 0.30	0.026*
Kidney	17.90 ± 0.81	7.49 ± 0.17	<0.001*	6.75 ± 1.31	3.29 ± 0.81	0.018*	4.50 ± 0.89	2.57 ± 0.16	0.060
Blood	7.38 ± 0.14	5.94 ± 1.24	0.180	1.53 ± 0.03	1.58 ± 0.31	0.811	1.01 ± 0.44	1.30 ± 0.21	0.361
Muscle	2.71 ± 0.18	6.03 ± 0.72	0.011*	1.13 ± 0.03	2.04 ± 0.37	0.013*	0.81 ± 0.11	1.40 ± 0.45	0.088
Tumor	3.61 ± 0.22	4.45 ± 0.56	0.072	2.51 ± 0.99	2.78 ± 0.56	0.705	2.16 ± 0.24	2.26 ± 0.94	0.862
Tumor/Blood Ratio	0.49 ± 0.03	0.76 ± 0.05	0.002*	1.63 ± 0.51	1.76 ± 0.12	0.743	2.32 ± 0.53	1.70 ± 0.39	0.253
Tumor/Muscle Ratio	1.33 ± 0.10	0.74 ± 0.01	0.013*	2.21 ± 0.70	1.37 ± 0.16	0.174	2.67 ± 0.08	1.58 ± 0.24	0.004*

*p < 0.05.

### MicroPET Imaging Study

**Figure 6:**
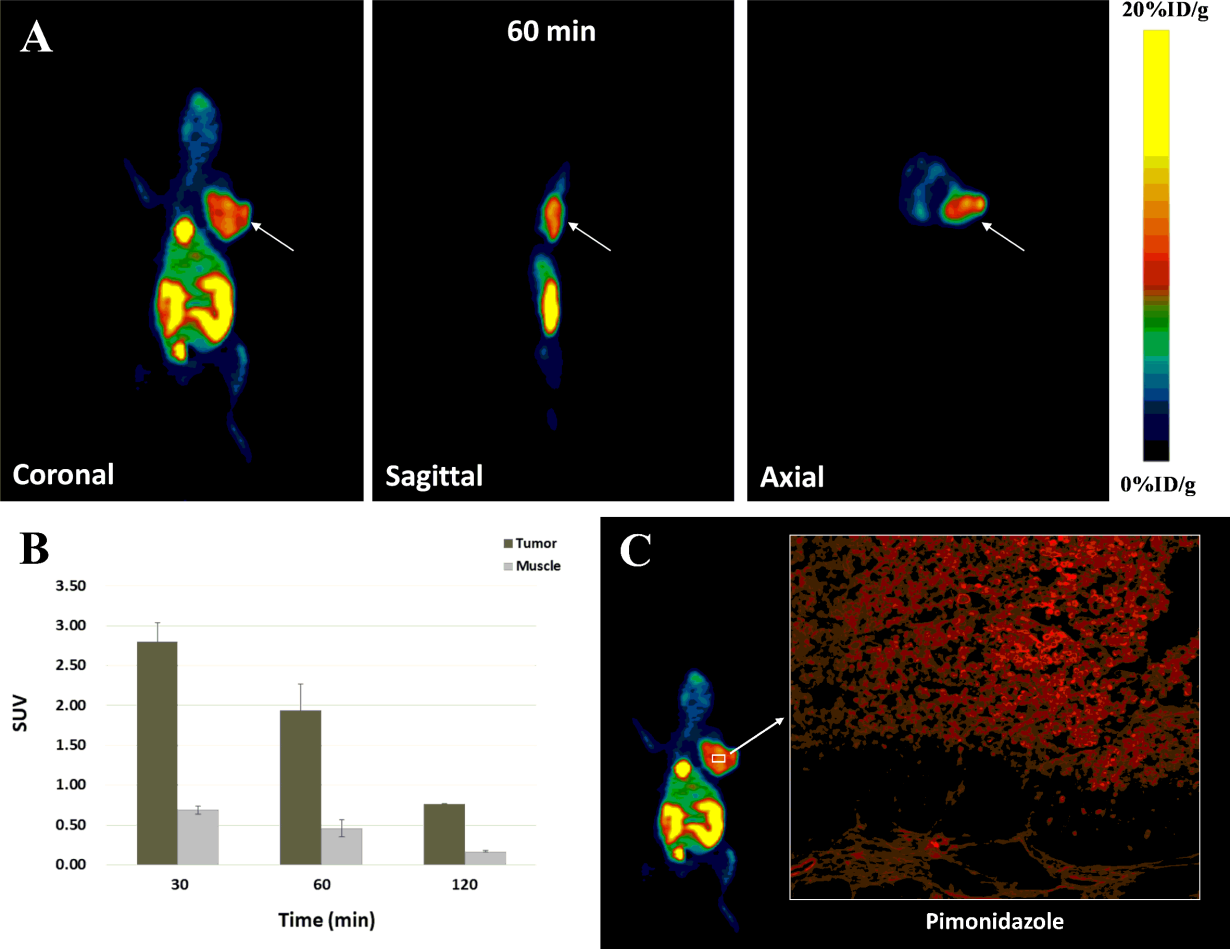
**(A)** MicroPET images of Al^18^F-NOTA-NI for tumor-bearing mice at 60 min after radiotracer administration (white arrows indicate tumors). **(B)** ROI-derived %ID/g for tumor and muscle, respectively (n = 4 per time point). **(C)** Immunofluorescence images of Pimonidazole in tissue sections of xenograft tumors (red).

MicroPET imaging studies were also conducted in ECA109 xenografted mice. Images were obtained from a two-hour dynamic scan and a series of static images were obtained at 30, 60 and 120 min after injection (**Figures 6A, B**). In the dynamic scan, the uptake of Al^18^F-NOTA-NI in kidney was higher than that in liver and decayed with time, whereas radiotracer was accumulated in the bladder and intestine since 20 min p.i. The peak value of imaging ROI-derived SUV for tumor was 2.80 ± 0.24 at approximately 30 min, and the tumor region was visualized with good tumor-to-muscle contrast as early as 30 min p.i., corresponding to the findings of the biodistribution study. In static images, T/M SUV ratios were 4.09 ± 0.59, 4.37 ± 0.94 and 4.53 ± 0.20 at 30, 60, and 120 min, respectively. By pimonidazole labeling and immunofluorescence imaging, we detected hypoxic regions in xenograft tumors (**Figure 6C**).

## Discussion

Rasey et al. assessed pretreatment hypoxia in a variety of tumors using ^18^F-MISO PET (17). In their study, hypoxia was heterogeneously distributed in 97% of the tumors studied, and tumor fractional hypoxic volume (FHV) (range 0 to 94.7%) did not correlate with the size, histology, or site. Hypoxic imaging is mainly used to detect tumor regions with hypoxia-related resistance to assess the prognosis. Qian et al (18). stated that the presence of hypoxia in imaging was associated with worse local recurrence, with a cumulative incidence of local recurrence at 12 months of 0% for patients without hypoxia versus 30% for patients with hypoxia (P <0.01). Since re-oxygenation strongly affects radiotherapy, the detection of hypoxia is needed to predict radiosensitivity, evaluate the therapeutic effect, and delineate the target volume. Generations of hypoxic tracers have been subjected to clinical tests and used in applications that lay the foundation for biological target volume (BTV) and biological intensity-modulated radiotherapy (BIMRT) (19–23). ^18^F-MISO, a classical nitroimidazole agent, has the disadvantage of requiring a complex labeling method and the pharmacokinetics (tumor-nonspecific accumulation and washout) cause background impurities. To explore more efficient production processes and more sensitive hypoxic tracers, we designed and synthesized a novel nitroimidazole agent, Al^18^F-NOTA-NI. In this compound, we added an amide bond at the linker between nitroimidazole and NOTA to produce a more hydrophilic agent. NOTA-NI was then labeled by ^18^F with high efficiency in one step at 100°C for 20 min. Our study showed that this tracer was stable at room temperature in PBS and at 37°C in human serum.

Esophageal cancer is an aggressive tumor with a poor prognosis and the 5-year survival rate for unresectable patients with concurrent chemoradiotherapy remains less than 20% (24). Overexpression of hypoxia-inducible-factor 1α (HIF-1α) strongly influence both tumor proliferation and lymph node metastasis in ESCC (25). So hypoxic detection was performed in esophageal carcinoma cell lines and xenografts. In vitro, we observed 2.27-fold greater contrast enhancement in hypoxic compared to normoxic esophageal squamous carcinoma cells ECA109, suggesting that this novel tracer is highly sensitive for detecting hypoxic tissue.

In contrast to the results in the *in vitro* uptake study, which depended on the oxygen partial pressure, in *in vivo* studies, the tracer’s efficiency could be influenced by factors such as biodistribution and elimination. Hydrophilic agents have generally been demonstrated to exhibit reduced liver uptake and increased kidney excretion. The introduction of amide bonds would make the product more negatively charged and hence may facilitate rapid clearance through the kidneys (26). The biodistribution study showed that the kidneys had the highest uptake of Al^18^F-NOTA-NI (17.90 ± 0.81%ID/g) 20 min p.i., while the liver had the highest concentration of ^18^F-MISO (9.16 ± 0.81%ID/g). After the distribution of the drugs stabilized in each tissue, the tumor-to-kidney uptake ratios for Al^18^F-NOTA-NI and ^18^F-MISO were 0.48 ± 0.05 and 0.88 ± 0.37, respectively. The tumor to liver and intestines ratios were 0.36 ± 0.10 and 0.28 ± 0.12, respectively. ^18^F-MISO was eliminated mostly through the entero-hepatic pathway, Al^18^F-NOTA-NI was mainly washed out *via* the kidneys. Notably, the high concentration in the bladder and the low uptake in the intestine would obscure masses located in the abdominal area, while the tumor-to-nontumor (liver) ratios gradually increased. Due to its hydrophilicity, Al^18^F-NOTA-NI hardly diffused into cells and showed lower uptake than ^18^F-MISO in tumors, as well as in normal organs. The initial uptake values for the novel tracer in tumors and other normal tissues were 3.61 ± 0.22%ID/g and 48.00 ± 1.52%ID/g, while the values for ^18^F-MISO were 4.45 ± 0.56%ID/g and 62.80 ± 2.17%ID/g. At two hours p.i., the uptake values for Al^18^F-NOTA-NI in tumors and normal tissues decreased to 2.16 ± 0.24%ID/g and 15.84 ± 1.20%ID/g, and the values for ^18^F-MISO fell to 2.26 ± 0.94%ID/g and 21.39 ± 0.71%ID/g, respectively. In this period of time, the uptake values in normal tissues for Al^18^F-NOTA-NI and ^18^F-MISO had statistically significant difference (*p* = 0.002), but there was no difference in tumor uptake values (*p* > 0.05). The novel tracer was eliminated more rapidly from surrounding tissues but was retained in hypoxic tumor cells, resulting in higher tumor-to-blood and tumor-to-muscle ratios. We analyzed the results by students t-test and demonstrated T/M ratio for Al^18^F-NOTA-NI was statistically higher than that for ^18^F-MISO, but the difference of T/B ratios had no statistical significance. Considering the small number of experimental mice, we will further expand the sample size to confirm the imaging characteristics of the new drug. Our results in PET imaging studies with ECA109 xenografted mice demonstrated that promising contrast between tumors and normal tissues appeared at 60 min after injection, and T/M SUV ratios continued to rise for two hours p.i. We have proved the presence of hypoxic regions in xenograft tumors by pimonidazole probe. Subsequently, we will further contrast the PET images with the immunofluorescence images on the same cross-section of the tumor, so as to estimate the correlation between the intratumoral uptake of the novel tracer and the hypoxia degree in a single xenograft tumor.

To improve the signal-to-background ratio of ^18^F-MISO PET, alternative hypoxia tracers, such as ^18^F-flortanidazole (^18^F-HX4), have been proposed and subjected to preclinical and clinical trials. ^18^F-HX4 is more water-soluble than ^18^F-MISO (logP: -0.69 vs logP: -0.4) (27). In Carlin et al.’s comparative study in animal models, tumor uptake of ^18^F-HX4 appeared to be broadly similar to that of ^18^F-MISO, but with more prominent renal uptake and less liver accumulation at this time point (28). Wack et al (29). demonstrated that ^18^F-HX4 showed a six-fold higher clearance than ^18^F-MISO in clinical tests. Although the absolute tracer activity for ^18^F-HX4 was lower at four hours p.i., ^18^F-HX4 showed significantly higher median contrast (2.08, range 1.87-2.73) over all patients than ^18^F-MISO (1.58, range 1.54-1.64). Another study in head and neck squamous cell carcinoma (HNSCC) patients showed that ^18^F-HX4 had faster clearance and a shorter injection-acquisition time, to give a T/M ratio similar to that of traditional ^18^F-MISO (1.5 h vs. 2 h p.i.) (30). The peculiarities of ^18^F-HX4, as a hydrophilic drug, were in line with those of our tracer. We found that the contrast ratios for all tracers continued to increase over time, and an increase in hydrophilicity accelerated the appearance of obvious contrast between hypoxic tumors and nontarget tissues, due to the faster elimination of free tracer and the resulting decrease in background signal. However, we should not ignore the fact that nitroreductase, the target of nitroimidazole derivatives, is an intracellular enzyme. More hydrophilic tracers tend to have lower tumor uptake because there is insufficient agent to freely diffuse into cells. Therefore, we sought to weigh the pros and cons of reducing lipophilicity. The current experimental results showed that Al^18^F-NOTA-NI had an ideal detection result in this aspect and significantly improved the contrast between tumor and background compared with ^18^F-MISO. We can further compare it with ^18^F-HX4 which is more hydrophilic.

A study indicated that fluorinated nitroimidazoles showed increased radiotracer uptake with not only pimonidazole but also CAIX staining, compared to the use of ^64^Cu-ATSM to observe hypoxic regions with low staining (28). Dubois et al (31). showed that ^18^F-HX4 imaging was highly correlated with the endogenous hypoxic marker CAIX so that it could reflect the tumor hypoxic state more accurately than ^18^F-MISO. Further studies should perform immunofluorescence staining to evaluate the relationship between novel tracer distribution and the mechanism of hypoxia. Furthermore, additional preclinical research and clinical applications should be carried out to better understand individual variations in clearance and distribution.

## Conclusions

We designed and synthesized an ^18^F-radiolabeled 2-nitroimidazole derivative conjugated with the bifunctional chelating agent NOTA. We testified the radiochemical purity, stability, uptake by hypoxic cells, and target-to-background ratio in *in vitro* and *in vivo* experiments. This imaging agent was principally excreted *via* the kidneys and xenografted tumors were distinguished from para-carcinoma tissue. Our results revealed that this hydrophilic tracer was suitable for hypoxia imaging due to its outstanding pharmacokinetic properties, such as ideal infiltration into hypoxic tumors, fast clearance of free tracer and low uptake into normal tissues.

## Data Availability Statement

The original contributions presented in the study are included in the article/supplementary materials, further inquiries can be directed to the corresponding authors.

## Ethics Statement

The animal study was reviewed and approved by the ethics committee of Nanjing Medical University.

## Author Contributions

X-CS is the corresponding author that contributed to design and guide of this research. QZ is the corresponding author too. JL is the first author that contributed to synthesis the novel tracer and write this paper. CZ contributed to the experiments *in vitro* and vivo. XY contributed to 18F labeling. X-JY contributed to the experiments *in vitro* and vivo. All authors contributed to the article and approved the submitted version.

## Funding

This study was funded by grants from National Natural Science Foundations of China (nos. 81703027, 81703028, and 81672983) and Science and Technology Department of Jiangsu Province (no. SBE2016740780). We acknowledge all the investigators who participated in this study.

## Conflict of Interest

The authors declare that the research was conducted in the absence of any commercial or financial relationships that could be construed as a potential conflict of interest.
